# Editorial: Immune system interactions in hematological tumor microenvironments: pathways to innovative treatments

**DOI:** 10.3389/fimmu.2025.1664847

**Published:** 2025-08-01

**Authors:** Elisa Mandato, Andrea Visentin

**Affiliations:** ^1^ Department of Medical Oncology, Dana-Farber Cancer Institute, Boston, MA, United States; ^2^ Hematology Unit, Department of Medicine, University of Padova, Padua, Italy

**Keywords:** hematological malignancies, tumor micro environment (TME), immune suppression, therapy, immune evasion

Blood cancer cells exploit multiple strategies to rewire immune cell compartments in the tumor microenvironment (TME) and avoid recognition and eradication by the immune system. Reciprocal interactions between tumor and immune cells control malignant cell survival and growth as well as resistance to treatment and immune evasion (1–7). A deep understanding of the specific TMEs in leukemia, lymphoma and multiple myeloma is necessary to develop novel effective therapeutic strategies.

This Research Topic includes a collection of innovative studies and reviews focused on the characteristics of the TME and the therapeutic approaches adopted in various hematological malignancies. Our aim is to enhance the current understanding of the roles of the TME in these malignancies and define how immune cells can be leveraged for more effective immunotherapies.

A comprehensive analysis of malignant cells’ genome has become the standard approach to recognize new disease *loci* and will eventually guide therapeutic decisions. Li et al. demonstrated the existence of specific single nucleotide polymorphisms (SNPs) in immunosuppression-related genes (*CIITA*, *CD200*, *CD163*, *MRC1* and *LILRB4*) in a cohort of over 300 acute myeloid leukemia (AML) patients. These SNPs were associated with AML susceptibility, sensitivity to treatment, overall survival and abnormal karyotype. The study evidenced that these genes were overexpressed in AML patients compared to healthy donors and provides important reference points for guiding clinical treatment and predicting outcomes in AML patients.

One of the major strategies adopted by cancer cells to avoid immune cell-mediated recognition and destruction is to foster the generation of an immune suppressive microenvironment. Vom Stein et al. provided a comprehensive perspective on the TME composition in chronic lymphocytic leukemia (CLL) and Richter Transformation (RT) that became well understood after the generation and analysis of patient-derived and genetically engineered mouse models. After a detailed description of the immune-suppressive TME in CLL and RT, the authors summarized the current challenges and opportunities of chimeric antigen receptor (CAR)-T, immune checkpoint inhibitors and bispecific antibodies for the therapy of these diseases.

CAR-T cell therapies have improved the outcome of patients with relapsed/refractory hematological malignancies; however, the complexity and safety concerns of this approach have paved the way for the evaluation of alternative strategies. CAR-NKs have recently yielded promises in the immunotherapy of many hematological malignancies (8). Yang et al. discussed the features and functions of NK cells, the construction of CAR-NKs and the advantages of CAR-NK over CAR-T cells. The authors also described the clinical employment of CAR-NKs in leukemias, lymphomas and multiple myeloma evidencing the current limitations and the strategies to overcome them in clinical practice.

The heterogeneity of the TME associated with its immunosuppressive properties is a major obstacle to reaching an effective immune-mediated anti-tumor activity. Kaweme et al. provided a detailed overview of the current sources of NK cells and the ongoing interventions to enhance their functions, circumvent the immunosuppressive TME in AML and chronic myeloid leukemia (CML) and improve the efficacy of NK cell-based immunotherapies in these malignancies.

Several patients with high-risk and relapsed/refractory hematological malignancies require hematopoietic stem cell transplantation (HSCT) (9) Graft versus leukemia is a critical component of a successful transplantation and involves donor cells eradicating leukemic cells within the recipient. Minculescu et al. analyzed the effects of granulocyte colony-stimulating factor (G-CSF), the preferential mobilizing agent in healthy donors, on TCR γδ and NK cells in the blood of healthy donors. The study evidenced that G-CSF stimulation in healthy donors for HSCT mobilized TCR γδ similarly to αβ cells and preferentially mobilized the nonVδ2 type. G-CSF also preserved NK cells of the CD56^dim^ subset and increased the expression of the activating receptor NKG2D both of which have been associated with anti-tumor effects.

Due to the depletion of B cells, infectious complications are a major cause of morbidity and mortality in B-cell hematological malignancies. Trained immunity-based vaccines have shown a promising strategy in patients with recurrent infections. Ochoa-Grullon et al. assessed the clinical benefit of MV130 in 15 hematological malignancy patients with recurrent respiratory tract infections. The study evidenced decreased infection rate and enhanced humoral immune responses.

In line with these findings, Yu et al. and Chen et al. explored novel treatments for patients with T cell non-Hodgkin lymphoma. Yu et al. investigated CMOEPs (cyclophosphamide, mitoxantrone hydrochloride liposome, vincristine, etoposide, and prednisone) in treatment naive patients with peripheral T lymphoma. No patients experienced dose-limiting toxicities and the recommended phase 2 dose of liposomal mitoxantrone was 18 mg/m^2^. No deaths due to toxicities were reported. Among the 12 patients evaluated for best response, the rate of complete remission (CR) was 66.7% and after a median follow-up only 2 patients died after disease progression. Chen et al. investigated VRMP (bortezomib, lenadomide and methylprednisolone) and ciclosporin A plus interferon-α as third line therapy in subcutaneous panniculitis-like T-cell lymphoma. The study evidenced that these chemo-free regimes led to longer time to next treatment than conventional chemotherapy.

The novel approaches targeting also the TME (liposomal mitoxantrone on tumor associate macrophages, ciclosporin A on T cells, etc.) appear promising and warrant further validations.
